# Chiral Separation of rac-Propylene Oxide on Penicillamine Coated Gold NPs

**DOI:** 10.3390/nano10091716

**Published:** 2020-08-30

**Authors:** Nisha Shukla, Zachary Blonder, Andrew J. Gellman

**Affiliations:** 1 Institute of Complex Engineered Systems, Carnegie Mellon University, Pittsburgh, PA 15213, USA; nisha@andrew.cmu.edu; 2 Department of Chemical Engineering, Carnegie Mellon University, Pittsburgh, PA 15213, USA; zblonder@andrew.cmu.edu; 3 W.E. Scott Institute for Energy Innovation, Carnegie Mellon University, Pittsburgh, PA 15213, USA

**Keywords:** chiral, nanoparticles, enantioselective adsorption, penicillamine, cysteine

## Abstract

The surfaces of chemically synthesized spherical gold NPs (Au-NPs) have been modified using chiral L- or D-penicillamine (Pen) in order to impart enantioselective adsorption properties. These chiral Au-NPs have been used to demonstrate enantioselective adsorption of racemic propylene oxide (PO) from aqueous solution. In the past we have studied enantioselective adsorption of racemic PO on L- or D-cysteine (Cys)-coated Au-NPs. This prior work suggested that adsorption of PO on Cys-coated Au-NPs equilibrates within an hour. In this work, we have studied the effect of time on the enantioselective adsorption of racemic PO from solution onto chiral Pen/Au-NPs. Enantioselective adsorption of PO on chiral Pen/Au-NPs is time-dependent but reaches a steady state after ~18 h at room temperature. More importantly, L- or D-Pen/Au-NPs are shown to adsorb R- or S-PO enantiospecifically and to separate the two PO enantiomers from racemic mixtures of RS-PO.

## 1. Introduction

Chirality has attracted enormous interest in the field of chemistry due to the enantiospecific responses of living organisms to the two enantiomers of chiral compounds that they have ingested. These differences in response are important in pharmaceutical industries because it is often the case that only one enantiomer of a chiral drug is therapeutically active. While one enantiomer of a drug can treat disease, the other enantiomer can have fatal or long-lasting side effects. Consequently, chiral pharmaceuticals that are produced synthetically must be prepared in enantiomerically pure form. The same is true of other chiral bio-active chemicals such as agrochemicals, pesticides and flavors. In recent years, researchers have also focused on the role of chirality in other areas such as catalysis [1], chiroptical applications [2,3], chiral magnetism [4], chiral semiconductors [5], cellular imaging [6], sensing [7], biomedical applications [8], chiral molecular assembly on surface [9], supramolecular chirality [10] and synthesis of chiral nanoparticles (NPs) [11]. One emerging field is chiral luminescent nanomaterials which have potential applications in chiral sensing [7]. Although a number of these studies have required synthesis of nanomaterials, it is still challenging to synthesize well-defined, monodispersed and inherently chiral NPs.

In previous work, we have shown that L- and D-cysteine (Cys, HO_2_CCH(NH_2_)CH_2_SH) modified surfaces of Au-NPs are capable of enantiospecific adsorption of racemic propylene oxide (PO) [11,12] and the chiral pharmaceutical propranolol hydrochloride [13]. In these studies, Au-NPs were either spherical or tetrahexahedral (24-sided) in shape. A systematic study has also been performed to understand the effects of temperature and of Au-NP size on enantiospecific adsorption from racemic PO [14]. These studies made use of optical polarimetry as a means of detecting enantioselective adsorption and used a model developed by us to analyze optical rotation measurements and quantify enantiospecific adsorption equilibrium constants.

**Figure 1:**
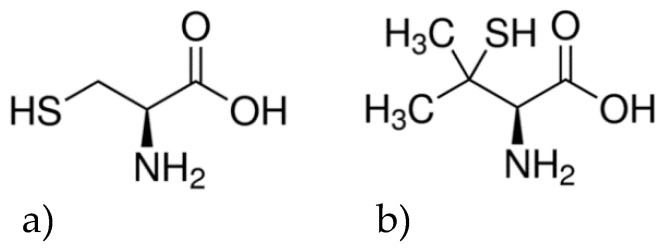
The molecular structures of (**a**) L-Cys and (**b**) L-Pen differ only in the addition of two methyl groups to the terminal carbon.

In this work, we have used spherical Au nanoparticles, which were synthesized based on our previous published work [11]. We chose to use spherical Au nanoparticles based on our previous work [14], which showed that the size of Au-NPs has an influence on their adsorption of chiral compounds and subsequent optical rotation. Smaller sized chiral Au-NPs yield higher optical rotation during exposure to racemic mixtures of chiral probe molecules. This indicates that smaller chiral NPs yield more effective enantiomer separations than large NPs, presumably as a result of their higher surface area. In comparison to our past work, we have developed this work in two directions. First, we have changed the chiral surface modifier used to render the surfaces of Au-NPs enantioselective. Different chiral ligands have different optical rotations and different capacities for adsorption and interaction with chiral probe molecules. We have substituted the D-/L-Cys (Figure 1a) used as a chiral surface modifier in our past work [11,12,13] with D-/L-penicillamine (Pen, HO_2_CCH(NH_2_)C(CH_3_)_2_SH) (Figure 1b). In our notation, D-/L-denotes either pure enantiomer, while DL- denotes use of the racemic mixture. Penicillamine was chosen as chiral modifier because its chemical functionality and structure are similar to those of Cys. Both are amino acids with alkyl thiol side groups. Cys is one of the 20 naturally occurring chiral amino acids that is polar in nature and is the only naturally occurring amino acid with a thiol group (-SH) [15]. Adsorption of Cys on Au surfaces is dependent on the pH of the solution [16]. At an acidic pH, Cys bonds to the Au surface mainly via deprotonation of the -SH bond. Bonding via the carboxylic acid group or amine group is minimal, although the –NH_2_ group is converted to –NH_3_^+^. On the other hand, in alkaline pH, Cys still bonds with the Au surface mainly via -SH bond deprotonation but also via the –NH_2_ group. In alkaline pH, the carboxyl group is ionized, and some studies suggest that it is aligned parallel to the Au surface. Some DFT studies suggest that Cys can bond with the Au surface via both thiolate and amino-thiolate modes. The bond strength of N–Au and S–Au were calculated to be 6 and 47 kcal/mol, respectively [17]. Another difference between Pen and Cys is their specific optical rotation. The specific optical rotation of Pen in water is ~9× higher than that of Cys. Some studies have shown that Pen is a strong chelating agent and bonds with many metals, particularly with Cu [18,19,20].

The second direction in which this work expands on our prior accomplishments is by documenting the effect of adsorption time on enantioselective adsorption of racemic RS-PO on chirally modified Au-NPs. In the past, enantioselective adsorption was studied within one hour of exposure of the chiral NPs to the chiral probe PO [11,12,13,14]. Here, we find that the equilibration of PO on Pen/Au-NP requires ~18 h of exposure. Our work shows that when using Pen as a chiral ligand, only weak enantiomeric separation of PO is observed within one hour of PO exposure; however, there is an almost ten-fold increase in enantiomeric separation after 18 h of PO exposure.

## 2. Materials and Methods

### 2.1. Synthesis of Au Nanoparticles

Spherical Au-NPs were prepared using the synthesis described in our previous work [11,12]. Monodispersed Au-NPs were obtained by reduction of Au(III) chloride hydrate (HAuCl_4_⋅H_2_O) using sodium borohydride (NaBH_4_) in the presence of sodium citrate dihydrate (HOC(COONa)(CH_2_COONa)_2_⋅2H_2_O) at room temperature. A typical synthesis involves preparation of 200 mL of 2.5 × 10^−4^ M Au(III) chloride hydrate in deionized water. The solution was stirred at a constant rate for 5–10 min to obtain a light-yellow homogenous solution. Next, 0.015 g of sodium citrate dihydrate was added to the Au(III) chloride hydrate solution. The color of the solution changed from light yellow to colorless. Once the change in color was observed, the stirring speed was accelerated. Next, 6 mL of ice-cold 0.1 M sodium borohydride was slowly added to the reaction solution at a constant rate over a period of one minute. The accelerated stirring was continued for another minute and then the speed was turned to moderate stirring. The color of the reaction solution then turned to wine red. Finally, the reaction solution was allowed to stir for another 5 h. The Au-NP solutions were stored in glass vials and covered with aluminum foil to prevent degradation by light.

### 2.2. Coating of Au Nanoparticles with D- or L-penicillamine

Following synthesis, the Au-NP solution was diluted (3 mL of solution into 15 mL of deionized water), and 0.067 g of D- or L-Pen was added to the diluted Au-NP solution. The final concentration of D- or L-Pen was 0.025 M. The solution was allowed to sonicate for 10 min prior to use. Once the Au-NPs were coated with D- or L-Pen and then exposed to PO, the solutions were allowed to sit for 18 h before optical rotation measurements were taken. A wait time of 18 h was chosen after initial experiments observed that adsorption of PO on D- or L-Pen/Au-NPs reaches a steady state after 18 h.

### 2.3. Characterization of Au-NPs

**Figure 2:**
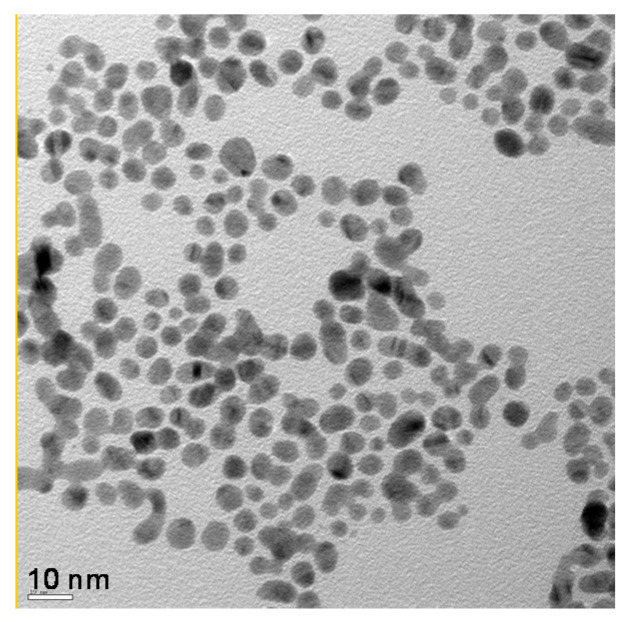
TEM image of Au-NPs. Nanoparticles are roughly spherical.

Transmission electron microscopy (TEM) imaging of the Au-NPs prepared using the procedure described above indicates that they are roughly spherical and about 3−5 nm in diameter (Figure 2). The Au-NPs were also characterized using UV-Vis spectroscopy. In order to obtain the UV-Vis spectra, dilute solutions of Au-NPs were scanned over the range 400−900 nm using a Cary UV-Vis spectrophotometer (Agilent, Santa Clara, CA, USA). A distinct peak of 513 nm was observed, which is characteristic of 3−5 nm spherical Au-NPs [4]. The full width at half maximum (FWHM) of the peak was also narrow, suggesting that the Au-NPs were fairly monodispersed.

### 2.4. Optical Rotation Measurements

Optical rotation measurements were carried out using a Rudolph Research Analytical Autopol VI (Hackettstown, NJ, USA). This polarimeter can measure optical rotation of samples at controlled temperatures and at six different wavelengths. In a typical optical rotation measurement, 3 mL of sample solution was put into a Temptrol polarimeter sample cell (Hackettstown, NJ, USA). All measurements in this work were carried out using 436 nm wavelength light and at a temperature of 23 °C.

The optical rotation measurement protocol focused on preparation of solutions with well-controlled fractional concentrations of R-, S- or RS-PO in solutions of Pen/Au-NPs. In a typical set of optical rotation measurements, 14 scintillation vials were used. Seven vials had 6 mL of L-Pen/Au-NPs and the other seven vials had 6 mL of D-Pen/Au-NPs. The solution was sonicated for 10 min. After sonication, 0, 10, 20, 30, 40, 60 and 80 µL of R- PO was added to seven vials of L-Pen/Au-NPs. The concentrations of PO in the vials were 0, 0.023, 0.047, 0.07, 0.09, 0.14 and 0.19 M. This procedure was repeated with D-Pen/Au-NPs. The solutions were then allowed to sit for 18 h to equilibrate R-PO adsorption. A similar protocol was used with S-PO and RS-PO. After 18 h, optical rotation of all solutions was measured using the temperature-controlled polarimeter.

Au(III) chloride hydrate (>99.9%), sodium borohydride, L-Pen (>99%), D-Pen (>99%) and sodium citrate dihydrate (99%) were purchased from Sigma Aldrich (St. Louis, MO, USA). R- and S-PO (>98%), and RS-PO (>98) were purchased from Alfa Aesar (Havervill, MA, USA). All chemicals were used without further purification.

## 3. Results

**Figure 3:**
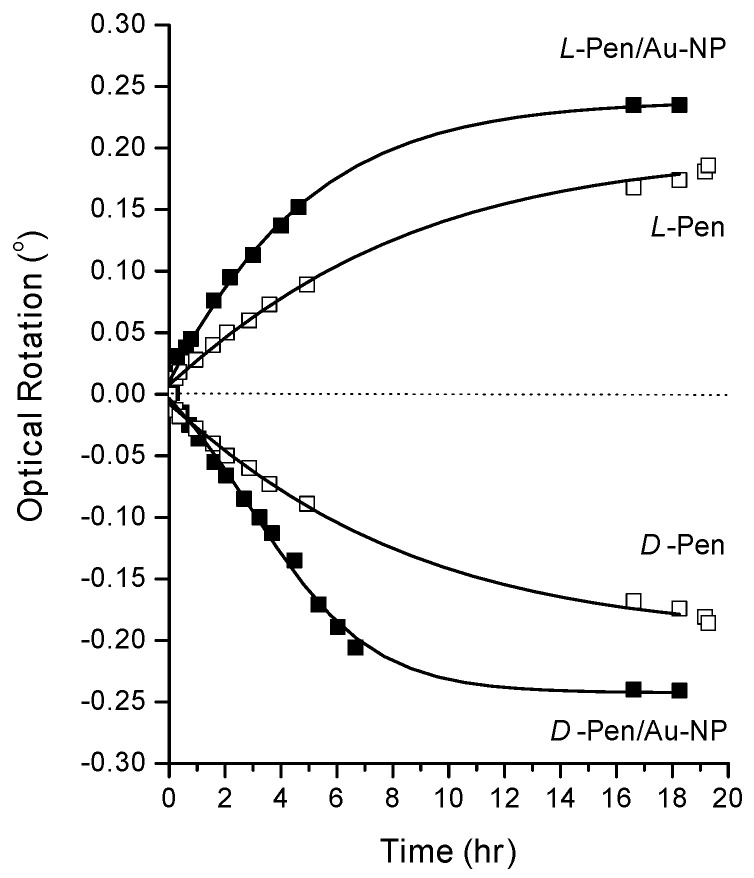
Optical rotation versus time by L- and D-Pen-coated Au-NPs and enantiomerically pure L- and D- Pen after exposure to a solution of 0.023 M RS-PO. The curves for D- and L-Pen are fits of a first-order exponential growth curve. The concentration of Au was 0.24 M and the concentration of Pen was 0.025 M.

All the enantiomeric separations and adsorption experiments were done after 18 h of Pen/Au-NP exposure to the PO probe. Figure 3 shows our rationale for using 18 h for PO probe adsorption. Figure 3 shows optical rotation by solutions of enantiomerically pure D- or L-Pen and D- or L-Pen/Au-NPs in solutions containing 0.023 M of RS-PO. The concentration of Au in these solutions is 0.24 M and the concentration of Pen is 0.025 M. Optical rotation measurements were taken at regular intervals over a period of ~20 h, as shown in Figure 3. As seen from the experiments, during the first 8 h the optical rotation changes significantly and at a roughly constant rate. However, after ~18 h of exposure to the RS-PO the optical rotations reach a steady state. One thing that is evident from these experiments is that the optical rotation of D- or L-Pen/Au-NPs is larger than that of enantiomeric pure D- or L-Pen.

Our detection of enantiospecific adsorption of chiral probes such as PO on chiral Au-NPs relies on measurements of the change in optical rotation observed during the addition of racemic RS-PO to solutions containing the chiral Au-NPs. In principle, the addition of a racemic probe to the solution should induce no change in optical rotation, unless that probe interacts enantiospecifically with the chiral Au-NPs such that one enantiomer adsorbs preferentially on the Au-NP surface. Detection of this separation by optical polarimetry also requires that the specific optical rotation constant of the adsorbed probe enantiomer differs from its value in solution [12,13].

**Figure 4:**
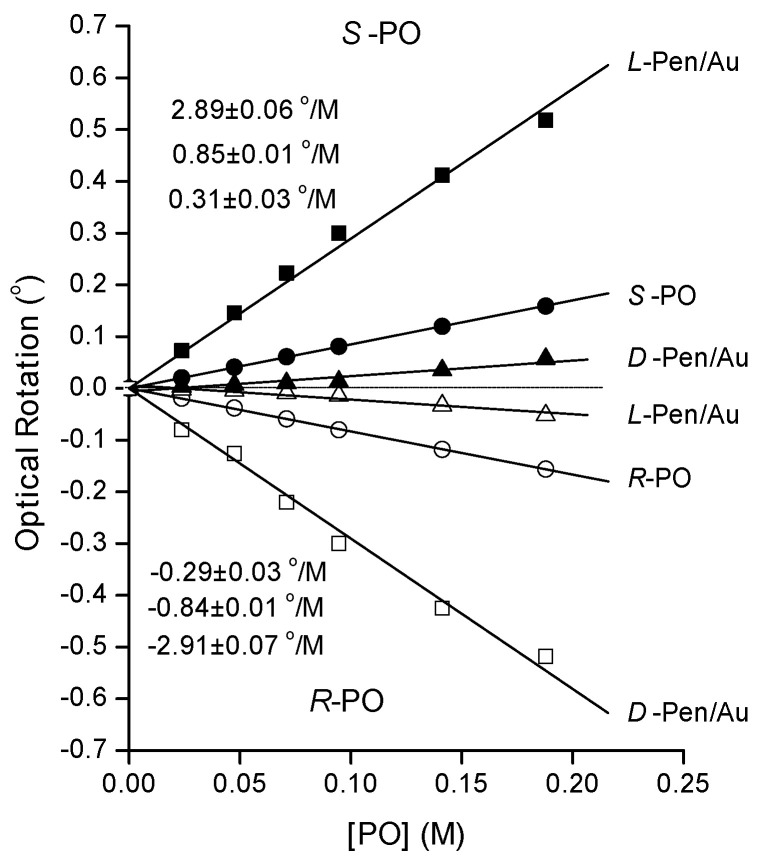
Optical rotation versus concentration of enantiomerically pure R- or S-PO in water and in solutions containing L- or D-Pen/Au-NPs. In aqueous solution, (● and ○), pure R- and S-PO rotate light with a specific rotation of ±0.85 ± 0.01 °/M. In the presence of L- or D-Pen/Au-NPs, the optical rotation for S-PO changes from 2.9 to 0.3 °/M. The concentration of Au was 0.24 M and the concentration of Pen was 0.025 M.

Prior to studying the optical rotation induced by adding racemic RS-PO to solutions containing chiral Au-NPs, we have studied the rotation induced by adding enantiomerically pure R- or S-PO to solutions containing D- or L-Pen-coated Au-NPs. Figure 4 shows plots of optical rotation as a function of the concentration of enantiomerically pure R- and S-PO. The data for S-PO are plotted with solid symbols and reveal positive optical rotations at all concentrations and in the presence of both D- and L-Pen/Au-NPs. It is clear that the PO enantiomer interacts enantiospecifically with the Au-NPs modified by the different enantiomers of Pen. Figure 4 provides clear evidence that some fraction of the enantiomerically pure PO in each solution must interact with the Pen/Au-NPs. As shown, the optical rotation magnitude for pure R- and S-PO is 0.85 ± 0.01°/M in close agreement with our prior observations [11]. The optical rotation magnitude for S-PO with L-Pen/Au-NPs is highest at ~2.9 ± 0.07 °/M. This is roughly 3.5× the specific optical rotation for enantiomerically pure S-PO. Note that, as required by diastereomerism, the magnitude of the optical rotation of R-PO in the presence of D-Pen/Au-NPs is also roughly 3.5× the rotation by pure R-PO. In contrast, S-PO with D-Pen/Au-NPs results in a suppression of optical rotation to 0.3 ± 0.03 °/M. This implies that S-PO interaction with the D-Pen/Au-NPs reduces and even inverts the optical rotation of S-PO. The same inversion is observed for the interaction of R-PO with L-Pen/Au-NPs.

**Figure 5:**
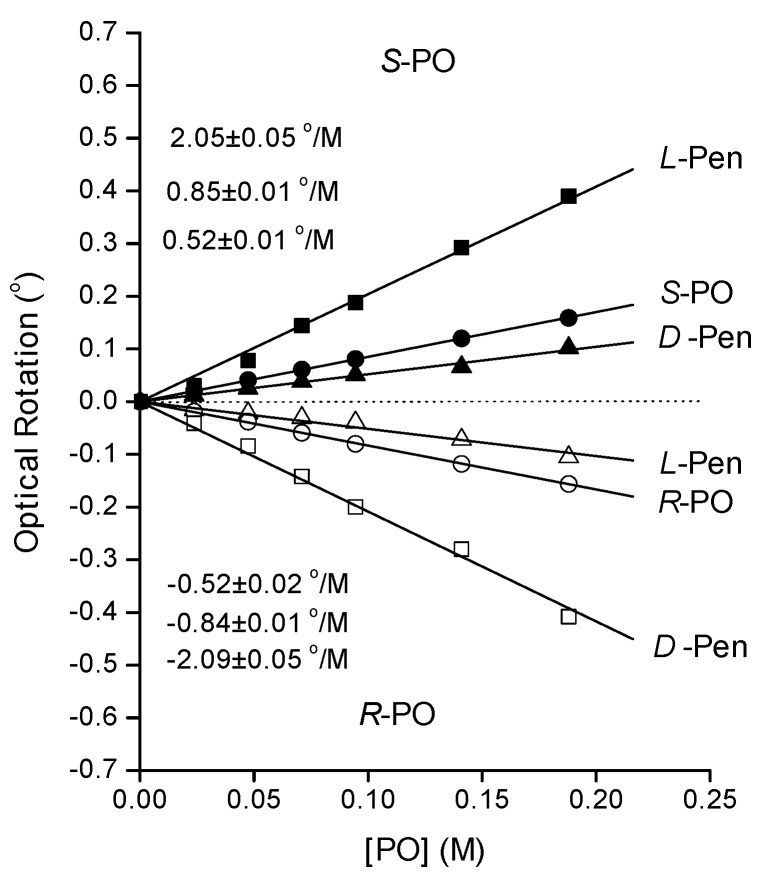
Optical rotation by enantiomerically pure R- and S-PO in water and in aqueous solutions of enantiomerically pure L- or D-Pen. In aqueous solution (● and ○), pure R- and S-PO rotate light with a specific rotation of ± 0.85 ± 0.01 °/M. In the presence of enantiomerically pure L- or D-Pen, the optical rotation of S-PO changes to 2.1 and 0.5 °/M, respectively. The optical rotations of R-PO are related to those of S-PO by diastereomerism. The concentration of D- and L-Pen was 0.025 M.

It is important to remember that the solutions containing Pen/Au-NPs also contain some free Pen enantiomers that are not adsorbed on the Au-NP surfaces. PO added to the solutions can, in principle, interact with both the Pen/Au-NPs and with the free Pen. To probe the magnitude of this effect, we conducted the same experiment as illustrated in Figure 4 but without the presence of Au-NPs. The results are shown in Figure 5. The magnitude of the optical rotations of S-PO with L-Pen and R-PO with D-Pen are ~2.1 ± 0.05 °/M, lower than the optical rotation observed for PO in solutions containing Pen/Au-NPs. It is important to point out that this differs from our prior work using Cys as the chiral surface modifier. PO shows no enantiospecific interaction with Cys in the solution phase [9]. In contrast, S-PO with D-Pen and R-PO with L-Pen show optical rotation magnitude of 0.5 ± 0.02 °/M which is greater than that observed for S-PO interacting with D-Pen/Au-NPs. The second set of experiments in Figure 5 uses R-PO as the probe and confirms the results obtained with S-PO. In both cases, the interactions with Pen/Au-NPs induce greater changes in magnitude of the optical rotations than observed with just the modifier in solution; i.e., the shift in optical rotation arises from the interaction of PO with both Pen/Au-NPs and Pen in solution.

**Figure 6:**
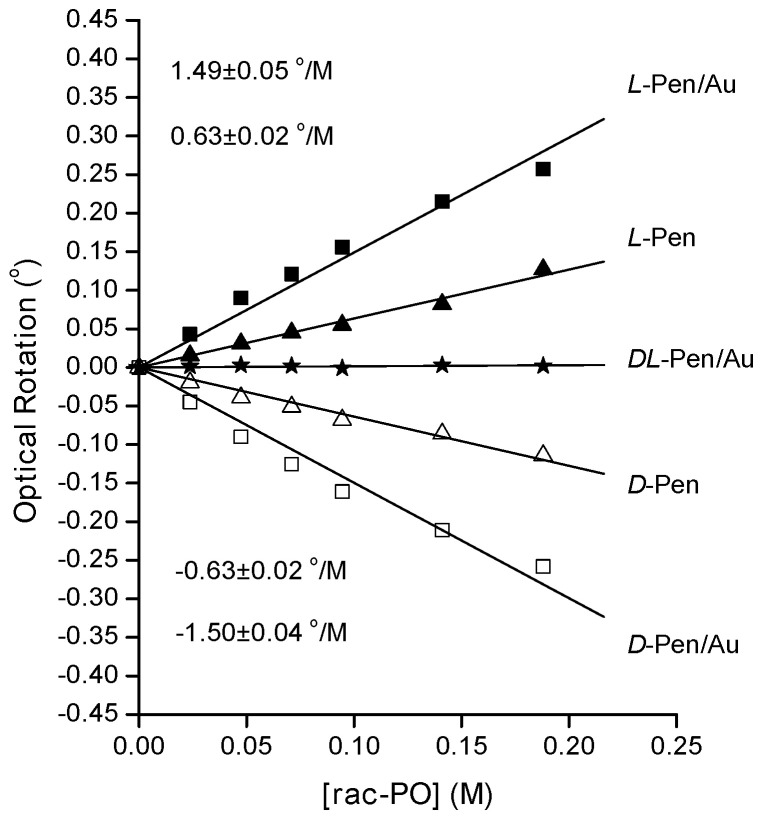
Optical rotation versus RS-PO concentration during addition to solutions containing Au-NPs coated with L-Pen (■), D-Pen (□) and DL-Pen (⋆). Addition of RS-PO to solutions containing enantiomerically pure D-Pen (Δ) and L-Pen (▲) indicates some interaction between PO and Pen. The concentration of Au was 0.24 M and the concentration of D-, L- and DL-Pen was 0.025 M.

The clearest demonstration of the enantiospecific interaction of the PO with chirally modified Pen/Au-NPs comes from measurement of optical rotation of light during addition of racemic RS-PO to solutions containing D- or L-Pen/Au-NPs. The results are shown in Figure 6. In the absence of enantiospecific interactions, the addition of a racemic compound to a solution would not be expected to cause any change in optical rotation. As Figure 6 shows, this is the case for addition of RS-PO to a solution containing racemic DL-Pen/Au-NPs. However, Figure 6 shows clear changes in optical rotation during addition of RS-PO to solutions containing either D- or L-Pen and during RS-PO addition to solutions containing either D- or L-Pen/Au-NPs. When RS-PO is added to a solution containing Au-NPs modified by DL-Pen at a concentration of 0.025 M, there is no net optical rotation, as both RS-PO and DL-Pen/Au-NPs have no net chirality. However, addition of RS-PO to the solution containing pure D-Pen/Au-NPs induced a rotation of −1.50 °/M (□), while addition of RS-PO to the solution containing pure L-Pen/Au-NPs induced a rotation of +1.49 °/M (■). The magnitudes of the optical rotation observed during addition of RS-PO to solutions containing D- or L-Pen/Au-NPs are almost twice the optical rotation observed during addition of RS-PO to solutions containing 0.025 M D- or L-Pen (Δ and▲, respectively) with no Au-NPs.

The structures of L-/D-Pen and L-/D-Cys are very similar, differing only in that Pen has two methyl groups attached to its side chain. However, enantioselective adsorption of PO on Cys- and Pen-coated Au-NPs differs in three major ways. The first is that Cys-coated Au-NPs show steady-state enantioselective adsorption of PO after one hour of exposure. However, in the case of Pen-coated Au-NPs we require 18 h of Au-NP exposure to the PO probe, in order to observe steady-state adsorption. This suggests that Cys reaches a steady-state coverage and conformation on Au much more rapidly than Pen.

The second difference between the two chiral modification schemes is the optical rotation intensity. Cys-coated Au-NPs have lower optical rotations for enantioselective adsorption of racemic PO than Pen-coated Au-NPs.

The third difference between Cys and Pen as chiral modifiers of Au/NP surfaces is clearly shown in Table 1. Table 1 compares the optical rotations of pure R-, S- and racemic PO on Cys- and Pen-coated Au-NPs. The optical rotations by R- and S-PO on L- or D-Cys-coated Au-NPs are almost identical. However, there is a significant difference in the optical rotation of R- and S-PO on L- or D-Pen-coated Au-NPs. S-PO has higher optical rotation with L-Pen-coated Au-NPs and lower on D-Pen-coated Au-NPs. On the other hand, R-PO shows the opposite effect. R-PO has higher optical rotation with D-Pen-coated Au-NPs and lower optical rotation L-Pen-coated Au-NPs.

## 4. Conclusions

Our work suggests that enantioselective adsorption of PO on Cys-modified Au-NPs is different than on Pen-modified Au-NPs. Enantiomerically pure R- or S-PO shows different optical rotations on D- or L-Pen-coated Au-NPs. On the other hand, enantioselective adsorption of pure R- or S-PO on D- or L-Cys-coated Au-NPs is the same. In addition, the specific optical rotation observed for RS-PO on D- or L-Pen-coated Au-NPs is 10× higher than during adsorption on D- or L-Cys-coated Au-NPs.

## Figures and Tables

**Table 1:** Specific optical rotation during addition of R-, S- and RS-PO to solutions of Au-NPs that have been chirally modified with D- or L-Pen, or D- or L-Cys.

Optical Rotation (°/M)	D-Pen/Au-NP	L-Pen/Au-NP	D-Cys/Au-NP	L-Cys/Au-NP
S-PO	0.31 ± 0.03	2.89 ± 0.06	1.69 ± 0.10	1.80 ± 0.06
R-PO	−2.91 ± 0.07	−0.31 ± 0.03	−1.66 ± 0.05	−1.75 ± 0.05
RS-PO	−1.50 ± 0.04	1.49 ± 0.05	0.15 ± 0.01	−0.15 ± 0.01
